# Prediction of neurologic deterioration based on support vector machine algorithms and serum osmolarity equations

**DOI:** 10.1002/brb3.1023

**Published:** 2018-06-11

**Authors:** Jixian Lin, Aihua Jiang, Meirong Ling, Yanqing Mo, Meiyi Li, Jing Zhao

**Affiliations:** ^1^ Department of Neurology Minhang Hospital Fudan University Shanghai China; ^2^ Emergency Department Minhang Hospital Fudan University Shanghai China; ^3^ Institute of Fudan‐Minhang Academic Health System Minhang Hospital Fudan University Shanghai China

**Keywords:** dehydration, neurological deterioration, support vector machine algorithm

## Abstract

**Objective:**

Dehydration on admission is correlated with neurological deterioration (ND). The primary objective of our study was to use support vector machine (SVM) algorithms to identify an ND prognostic model, based on dehydration equations.

**Methods:**

This study included a total of 382 patients hospitalized with acute ischemic stroke. The following parameters were recorded: age, sex, laboratory values (serum sodium, potassium, chlorinum, glucose, and urea), and vascular risk factor data. Receiver operating characteristic (ROC) curve analysis was used to evaluate the discriminative performance of the BUN/Cr ratio as well as each of 38 equations for predicting ND. We used the Boruta algorithm for feature selection. After optimizing the SVM kernel parameters, we built an SVM model to predict ND and used the test set to obtain predictive values for assessing model accuracy.

**Results:**

In total, 102 of 382 patients (26.7%) with acute ischemic stroke developed ND. In all patients, the BUN/Cr ratio and each of 38 equations were significant predictors of ND. Equation 20 [1.86 × Na+ + glucose + urea + 9] yielded the maximum area under the ROC curve, and faired best in terms of prognostic performance (a cutoff value of 284.49 mM yielded a sensitivity of 94.12% and specificity of 61.43%). Equation 32 predicted ND poststroke across population groups, and worked well in older as well as young adults; (a cutoff value of 297.08 mM yielded a sensitivity of 93.14% and specificity of 60.00%). Feature selection by the Boruta algorithm was used to decrease the number of variables from 18 to 5 in the condition. The specificity of test samples for the SVM prediction model increased from 44.1% to 89.4%, and the AUC increased from 0.700 to 0.927.

**Conclusions:**

SVM algorithms can be used to establish a prediction model for dehydration‐associated ND, with good classification results.

## 1 INTRODUCTION

Dehydration in patients with acute ischemic stroke has been observed in many clinical experimental studies (Crary et al., 2013). Furthermore, dehydration on admission is a strong predictor of clinical outcome and is correlated with the volume of the ischemic lesion and with an increased risk of mortality (Rowat, Graham, & Dennis, 2012; Schrock, Glasenapp, & Drogell, 2012). However, in the early assessment and treatment of cerebral infarction, serum osmolality is not regularly recommended as a diagnostic test (Adams et al., 2007). Therefore, the blood urea nitrogen (BUN)/creatinine (Cr) ratio, as a routinely available indicator of hydration, has been used in numerous studies (C. J. Lin et al., 2016; L. C. Lin, Lee, Hung, Chang, & Yang, 2014; Rowat et al., 2012; Schrock et al., 2012). Although use of the BUN/Cr ratio for evaluating dehydration has some value, it still has certain drawbacks. Defining the BUN/Cr ratio >15/1 as indicating dehydration is based on a priori knowledge. This prior knowledge is not entirely reliable and could affect results. Hence, it is necessary to determine a cutoff value of the BUN/Cr ratio that would yield the best diagnostic accuracy.

Serum osmolarity is associated with serum electrolyte and glucose levels (Siervo, Bunn, Prado, & Hooper, 2014). Consequently, electrolyte disturbances or diabetes status are likely to influence the accuracy of diagnosing dehydration (Stookey, Pieper, & Cohen, 2005). Therefore, a more precise marker for dehydration, based on the BUN/Cr ratio while also accounting for other factors, including electrolyte disturbances, and glucose metabolism disorders, is necessary. Furthermore, osmotically active determinants (serum sodium, potassium, urea, and glucose) should be used to derive a valid equation for the calculation of serum osmolarity.

Many equations have been used to calculate osmolarity (Fazekas et al., 2013; Siervo et al., 2014), but it remains unclear which equation performs best. It is also possible that a new formula could be devised that would better predict the functional outcome after an ischemic stroke.

Previously, logistic regression models have typically been used to analyze stroke outcome data. However, machine learning algorithms, which potentially have more powerful high‐level prediction performance, have been proposed as an alternative for analyzing large‐scale multivariate data (Bastanlar & Ozuysal, 2014). One of the most popular machine learning methods used for recognition or classification is the support vector machine (SVM) (Noble, 2006).

With the SVM, data are divided into a training set and a test set, the training dataset is used to build a classification algorithm model, which is then used to assign test set data to one or another category. The SVM algorithm has been widely applied in the biological sciences.

We aimed to design a dehydration equation that is not prone to the differential bias associated with the above‐mentioned factors, to improve diagnostic accuracy. The primary objective of our study was to use SVM algorithms to identify an ND prognostic model based on dehydration equations.

## 2 METHODS

### 2.1 Subjects

This retrospective study included a total of 382 patients hospitalized with acute ischemic stroke at Minhang Hospital Affiliated to Fudan University (Central Hospital of Minhang District in Shanghai), from January 2012 to June 2016. The study was approved by the Institutional Review Board of Minhang Hospital Affiliated to Fudan University.

### 2.2 Patients

This study included acute ischemic stroke patients who were admitted to the hospital within 7 days after symptom onset. This time window is appropriate for recruiting an adequate number of patients and for monitoring patients for ND. Using the World Health Organization (WHO) criteria, stroke was defined as relevant lesions on computed tomography (CT) or magnetic resonance imaging (MRI) (“Stroke‐‐1989. Recommendations on stroke prevention, diagnosis, and therapy. Report of the WHO Task Force on Stroke and other Cerebrovascular Disorders,” 1989). We defined ND as an increase of ≥2 points in the total National Institutes of Health Stroke Scale (NIHSS) score, an increase of ≥1 point on the level of consciousness or motor items of the NIHSS subscale scores, or the development of any new neurological deficits during the first 7 days of hospitalization (Weimar et al., 2005). ND was determined and validated by participating neurologists.

### 2.3 Data collection

We used a standardized data form to collect the participants’ data from a database. The following data were collected: age, sex, arterial blood pressure, and laboratory studies (serum sodium, potassium, chlorinum, glucose, and urea) on admission within the first 7 days. Information about vascular risk factors, such as hypertension, diabetes, hypercholesterolemia, ischemic heart disease, and smoking, was included in the collected database. We used the BUN/Cr ratio and 38 different equations to calculate serum osmolarity (Fazekas et al., 2013). The equations mostly involved summing multiples of serum sodium, potassium, glucose, and urea. The BUN/Cr ratio and 38 equations analyzed in this study are shown in Table 1.

**Table 1:** Demographic and clinical characteristics of patients with and without neurologic deterioration (ND)

	Male (*n *= 382)		*p* value
	ND absent	ND present	
Age, years	65.36 (10.29)	64.74 (9.76)	0.489
Sex, male	147 (52.5%)	52 (51.0%)	0.793
Time to treatment (hr)	44.4 (25.32)	42.57 (25.11)	0.816
Risk factors			
Hypertension	157 (56.1%)	52 (51.0%)	0.377
Diabetes	82 (29.3%)	34 (33.3%)	0.447
Atrial fibrillation	60 (21.4%)	23 (22.5%)	0.814
Hyperlipidemia	135 (48.2%)	55 (53.9%)	0.324
Current smoking	103 (36.8%)	34 (33.3%)	0.534
Coronary heart disease	172 (61.4%)	61 (59.8%)	0.773
Hyperhomocysteinemia	114 (40.7%)	48 (47.1%)	0.267
Laboratory studies			
Blood urea nitrogen (mg/dl)	14.08 (3.81)	20.86 (9.32)	0.001
Creatinine (mg/dl)	0.76 (0.24)	0.84 (0.45)	0.001
Sodium(Na) (mM)	141.67 (2.82)	144.38 (3.41)	0.5
Potassium(K) (mM)	3.63 (0.40)	3.66 (0.46)	0.302
Chlorinum (Cl) (mM)	103.33 (3.71)	105.23 (4.24)	0.419
Fasting plasma glucose (mM)	5.31 (1.53)	5.93 (2.12)	0.007
Systolic blood pressure (mmHg)	152.82 (30.87)	152.72 (27.96)	0.489

### 2.4 Statistical analysis

Continuous data were summarized as the mean (standard deviation). Normally distributed data and non‐normally distributed data were compared by the two‐sample *t* test, and Mann–Whitney U test, respectively. Categorical data were presented as number (percentage) and were compared by Pearson's chi‐square test or Fisher's exact test. Receiver operating characteristic (ROC) curve analysis was then used to evaluate the discriminative performance of the BUN/Cr ratio or each of 38 equations for predicting ND in patients. Specifically, ROC analysis was used to determine predictive performance by comparing the areas under the ROC curve (AUCs). All statistical assessments were two‐tailed, and statistically significant differences were indicated by a *p*‐value of <0.05. Statistical analyses were performed with SPSS 22.0 statistical software ( http://scicrunch.org/resolver/SCR_002865, http://www-01.ibm.com/software/uk/analytics/spss/).

### 2.5 Feature selection

Feature selection is a crucial step in predictive modeling (Weimar et al., 2005). Some features available in the study were relevant to ND, but may hinder the predictive model from achieving higher accuracy. Therefore, removing these variables from the model would increase prognostic accuracy. The Boruta algorithm is available in the R package and is useful for feature selection ( https://CRAN.R-project.org/package=Boruta). We used the Boruta algorithm to identify the most sensitive features and eliminate redundant features, biases, and unwanted noise.

### 2.6 SVM model

R language ( https://www.r-project.org/) is a processing environment for statistical computing and graphics. In this study, SVM classifiers were implemented using the e1071 package ( https://CRAN.R-project.org/package=e1071) in R. We applied SVM to the dataset to determine ND prognosis. The SVM model should be able to discriminate patients with ND and without ND.

The two datasets included 382 instances with 18 attributes for each instance. Of these, 56 attributes or 18 attributes were real‐value input features. There were 102 patients with ND, and 280 patients without ND in the dataset.

First, we randomly divided the dataset into two subsets, one with about 80% of the instances, for training, and another with around the remaining 20% of instances, for testing. Second, the two parameters (C‐ cost, r‐ gamma) could not be intuitively defined. However, the selection of these two parameters would affect the accuracy of an SVM model. Fortunately, the two parameters could be optimized via cross‐validation of the training data using the tune () function. Then, we implemented SVM classifiers with a radial basis function (RBF) kernel (exp (‐gamma*|u‐v|^2)). The convergence epsilon, which is an optimizer parameter used to specify the stop point for iteration, was set to 0.1. Finally, we built an SVM model and used the test set to obtain predictive values to assess the model accuracy. We also constructed an ROC curve and determined sensitivities and specificities for particular cutoff values.

## 3 RESULTS

### 3.1 Demographic and clinical characteristics of patients

Of 382 enrolled patients with acute ischemic stroke, 102 patients (26.7%) developed ND. There were no significant differences in age and sex between the patients with ND (52% male; mean age 64.72 ± 9.76 years) and without ND (52.5% male; mean age 65.36 ± 10.29 years). Moreover, there were no statistically significant differences between patients with and without ND in terms of a history of various risk factors: hypertension (*n *= 52, 51% vs. *n *= 147, 52.5%, *p *> 0.05), diabetes (*n *= 34, 33.3% vs. *n *= 82, 29.3%, *p* > 0.05), atrial fibrillation (*n *= 23, 22.5% vs. *n *= 60, 21.4%, *p* > 0.05), hyperlipidemia (*n *= 55, 53.9% vs. *n *= 135, 48.2%, *p* > 0.05), current smoking (*n *= 34, 33.3% vs. *n *= 103, 36.8%, *p* > 0.05), coronary heart disease (*n *= 61, 59.8% vs. *n *= 172, 61.4%, *p* > 0.05), and hyperhomocysteinemia (*n *= 48, 47.1% vs. *n *= 114, 40.7%, *p* > 0.05) in the study population. Similarly, mean systolic blood pressure (SBP) was not significantly different between the two groups (ND present: 152.82 ± 30.87 mmHg vs. ND absent 152.72 ± 27.96 mmHg, *p* < 0.05). In terms of laboratory tests, participants with ND differed significantly from those without ND in that they had higher BUN (20.86 ± 3.92 mg/dl vs. 14.08 ± 3.81 mg/dl, *p* < 0.05), creatinine (0.84 ± 0.45 mg/dl vs. 0.76 ± 0.24 mg/dl, *p* < 0.05), and fasting plasma glucose (5.93 ± 2.12 mM vs. 5.31 ± 1.53 mM, *p* < 0.05) concentrations. Table 1 summarizes the clinical and demographic characteristics of the patients.

### 3.2 Markers for the assessment of dehydration

There was a significant difference (*p* < 0.05) in the mean values of the BUN/Cr ratio, and the outcomes of Equation 1, Equation 3, Equation 6, Equation 11, Equation 13, Equation 16, Equation 17, Equation 18, Equation 19, Equation 20, Equation 22, Equation 23, Equation 25, Equation 25a, Equation 26, Equation 27, Equation 27a, Equation 28, Equation 29, Equation 30, Equation 31, Equation 32, Equation 33, Equation 34, and Equation 35 at admission between patients with and without ND. A persistent increase in the mean values of BUN/Cr ratio and other markers mentioned above was observed in patients with ND as compared to those without ND (Table 2) (Edelman, Leibman, O'Meara, & Birkenfeld, 1958).

**Table 2:** BUN/Cr ratio and calculated osmolarity (mM) in patients with acute ischemic stroke, stratified by neurologic deterioration (ND)

	ND absent	ND present	*p* value	Formula	References
BUN/Cr ratio	19.27 (5.28)	26.38(7.52)	0.001		
Equation 1, mM	265.72 (5.04)	272.41 (7.40)	0.029	1.75 × Na+ + glucose + 0.5 × urea + 10.1	Edelman et al. (1958)
Equation 2, mM	307.20 (7.41)	314.31 (8.97)	0.5	2.63 × Na+ − 65.4	Edelman et al. (1958)
Equation 3, mM	271.20 (5.33)	278.19 (7.74)	0.035	1.86 × Na+ + glucose + 0.5 × urea	Holmes (1962)
Equation 4, mM	298.30 (5.82)	305.73 (8.09)	0.053	2 × (Na++K+) + glucose + 0.5 × urea	Jackson and Forman (1966), Gerich, Martin, and Recant (1971)
Equation 5, mM	283.35 (5.64)	288.75 (6.82)	0.5	2 × Na+	Winters (1968)
Equation 6, mM	291.04 (5.70)	298.41 (8.19)	0.042	2 × Na+ + glucose + 0.5 × urea	Mahon, Holland, and Urowitz (1968)
Equation 7, mM	290.35 (5.64)	295.75 (6.82)	0.5	2 × Na+ + 7	Jetter (1969)
Equation 8, mM	293.34 (5.64)	298.75 (6.82)	0.5	2 × Na+ + 10	Ross and Christie (1969)
Equation 9, mM	288.52 (5.65)	294.68 (7.35)	0.167	2 × Na+ + glucose	Stevenson and Bowyer (1970)
Equation 10, mM	297.52 (5.92)	303.19 (7.16)	0.5	2.1 × Na+	Hoffman (1970)
Equation 11, mM	290.86 (5.70)	298.14 (8.12)	0.048	2 × Na+ + glucose + 0.93 × 0.5 × urea	Sadler (1970)
Equation 12, mM	293.82 (5.73)	301.14 (7.97)	0.053	(2 × (Na+ + K+) + glucose + 0.5 × urea) × 0.985	Gerich et al. (1971)
Equation 13, mM	276.20 (5.33)	283.19 (7.74)	0.035	1.86 × Na+ + glucose + 0.5 × urea + 5	Boyd and Baker (1971)
Equation 14, mM	290.34 (5.67)	297.55 (8.04)	0.052	2 × Na+ + 0.9 × glucose + 0.93 × urea × 0.5	Weisberg (1971)
Equation 15, mM	285.86 (5.69)	292.48 (7.63)	0.166	2 × Na+ + 0.5 × urea	Weisberg (1971)
Equation 16, mM	252.22 (4.96)	258.72 (7.20)	0.035	(1.86 × Na+ + glucose + 0.5 × urea)/0.93	Glasser, Sternglanz, Combie, and Robinson (1973)
Equation 17, mM	283.77 (5.54)	290.92 (7.78)	0.045	1.9 × (Na+ + K+) + glucose + 0.5 × urea	Wilson (1973)
Equation 18, mM	278.34 (5.30)	285.30 (7.71)	0.034	1.85 × Na+ + glucose + 0.5 × urea + 8.55	Dorwart (1973)
Equation 19, mM	280.20 (5.33)	287.19 (7.74)	0.035	1.86 × Na+ + glucose + 0.5 × urea + 9	Dorwart and Chalmers (1975)
Equation 20, mM	282.71 (5.47)	290.92 (8.83)	0.001	1.86 × Na+ + glucose + urea + 9	Dorwart and Chalmers (1975)
Equation 21, mM	298.12 (5.81)	305.46 (8.02)	0.065	2 × (Na+ + K+) + glucose + 0.93 × 0.5 × urea	Jenkins and Larmore (1974)
Equation 22, mM	291.01 (5.67)	299.47 (9.01)	0.001	1.89 × Na+ + 1.38 × K++ 1.08 × glucose + 1.03 × urea + 7.47	Bhagat, Garcia‐Webb, Fletcher, and Beilby (1984)
Equation 23, mM	290.47 (5.59)	298.73 (8.78)	0.001	1.86 × (Na+ + K+) + glucose + urea + 10	Bhagat et al. (1984)
Equation 24, mM	298.34 (5.67)	305.55 (8.05)	0.052	2 × Na+ + 0.9 × glucose + 0.93 × 0.5 × urea + 8	Snyder, Williams, Zink, and Reilly (1992)
Equation 25, mM	272.06 (5.37)	279.41 (8.05)	0.015	1.86 × Na+ + 1.03 × glucose + 1.28 × 0.5 × urea	Hoffman, Smilkstein, Howland, and Goldfrank (1993)
Equation 25a, mM	267.98 (5.29)	275.22 (7.93)	0.015	(1.86 × Na+ + 1.03 × glucose + 1.28 × 0.5 × urea) × 0.985	Hoffman et al. (1993)
Equation 26, mM	294.97 (4.44)	300.94 (6.78)	0.005	1.36 × Na+ + 1.6 × glucose + 0.45 × urea + 91.75	Wojtysiak, Duma, and Solski (1999)
Equation 27, mM	328.75 (5.84)	337.33 (9.26)	0.002	2 × Na+ + glucose + urea + 35.2	Koga, Purssell, and Lynd (2004)
Equation 27a, mM	323.82 (5.75)	332.27 (9.12)	0.002	(2 × Na+ + glucose + urea + 35.2) × 0.985	Koga et al. (2004)
Equation 28, mM	289.95 (5.43)	297.03 (7.86)	0.037	1.897 × Na+ + glucose + urea × 0.5 + 13.5	Rasouli and Kalantari (2005)
Equation 29, mM	288.77 (5.54)	295.92 (7.78)	0.045	1.9 × (Na+ + K+) + glucose + urea × 0.5 + 5	Rasouli and Kalantari (2005)
Equation 30, mM	280.47 (5.59)	288.73 (8.78)	0.001	1.86× (Na+ + K+) + glucose + urea	Varley, Gowenlock, and Bell (1980)
Equation 31, mM	294.32 (5.88)	303.02 (9.38)	0.002	2× Na+ + 1.15 × glucose + urea	Khajuria and Krahn (2005)
Equation 32, mM	295.24 (5.65)	303.62 (8.91)	0.001	1.86× (Na+ + K+) + 1.15 × glucose + urea + 14	Khajuria and Krahn (2005)
Equation 33, mM	297.43 (5.91)	306.09 (9.34)	0.002	1.09 × 1.86 × Na+ + glucose + urea	Bianchi, Bidone, and Arfini (2009)
Equation 34, mM	300.81 (5.96)	309.45 (9.20)	0.003	2 × (Na+ + K+) + glucose + urea	Wikipedia tfe (2014)
Equation 35 (tonicity), mM	295.78 (5.96)	309.45 (9.20)	0.258	2 × (Na+ + K+) + glucose	Stookey, Purser, Pieper, and Cohen (2004)

### 3.3 ROC curve of prediction of ND with various indicators of serum osmolarity

The prognostic accuracy for ND based on the BUN/Cr ratio and 38 equations in all patients are presented in Table 3. In all patients, the BUN/Cr ratio and each of the 38 equations were significant predictors of ND. An ROC curve can be used to illustrate and evaluate the accuracy of the classifier system. Sensitivity represents the probability that a true judgment is made for patients with ND, while specificity represents the probability that a true judgment is made for patients without ND. The AUC is the most commonly used precision index. The best possible prediction method would generate a value that would maximize the AUC. We therefore ranked the 38 equations including indicators of dehydration according to their AUC values. Equation 20 produced the maximum AUC of 0.849 (95% confidence interval (CI) [0.785–0.870], *p* = 0.000), which suggested that it had the highest ND prognostic performance. The optimal cutoff values were calculated using the Youden Index (maximum [sensitivity + specificity − 1]). The cutoff value with the highest sensitivity and specificity was 284.49 mM (sensitivity = 94.12%, specificity = 61.43%). In addition, an ROC curve was used to find the optimal cutoff point for the BUN/Cr ratio for the prediction of the occurrence of ND. The threshold value was 20.66 (AUC = 0.788, 95% CI [0.735–0.842], *p* = 0.000, sensitivity = 81.37%, specificity = 67.5%). These results are shown in Table 3. However, at least 10 equations had highly similar AUCs, ranging from AUC 0.849 (Equation 20) to AUC 0.841(Equation 34). Equation 32 performed well in older adults (Hooper et al., 2015; Siervo et al., 2014) and young adults (Heavens, Kenefick, Caruso, Spitz, & Cheuvront, 2014) and predicted poststroke ND across population groups, with a cutoff value of 297.08 mM (sensitivity = 93.14%, specificity = 60.00%).

**Table 3:** AUC, cutoff value, specificity, and sensitivity of prognostic accuracy of assessment of ND

	AUC	Cutoff value	Sensitivity (%)	Specificity (%)	Confidence interval (CI)		Rank
BUN/Cr ratio	0.788	20.66	81.37	67.50	0.735	0.842	31
Equation 1	0.830	267.55	88.24	63.21	0.806	0.886	13
Equation 2	0.748	308.72	76.47	57.86	0.788	0.872	34
Equation 3	0.827	273.22	88.24	63.57	0.694	0.801	15
Equation 4	0.823	300.94	81.37	66.79	0.785	0.870	23
Equation 5	0.748	284.50	76.47	57.86	0.779	0.866	35
Equation 6	0.825	293.16	87.25	63.57	0.694	0.801	20
Equation 7	0.748	291.50	76.47	57.86	0.782	0.868	36
Equation 8	0.748	294.50	76.47	57.86	0.694	0.801	37
Equation 9	0.779	289.78	85.29	56.43	0.694	0.801	33
Equation 10	0.748	298.73	76.47	57.86	0.731	0.828	38
Equation 11	0.823	292.95	87.25	63.21	0.694	0.801	25
Equation 12	0.823	296.42	81.37	66.79	0.780	0.866	24
Equation 13	0.827	278.22	88.24	63.57	0.779	0.866	16
Equation 14	0.821	292.42	87.25	62.14	0.785	0.870	27
Equation 15	0.793	286.93	89.22	55.71	0.778	0.865	30
Equation 16	0.827	254.10	88.24	63.57	0.745	0.842	17
Equation 17	0.825	285.35	89.22	59.64	0.785	0.870	21
Equation 18	0.828	280.30	88.24	63.21	0.782	0.868	14
Equation 19	0.827	282.22	88.24	63.57	0.785	0.870	18
Equation 20	0.849	284.49	94.12	61.43	0.785	0.870	1^a^
Equation 21	0.821	300.60	83.33	65.36	0.810	0.888	29
Equation 22	0.847	292.99	92.16	61.43	0.777	0.865	2^a^
Equation 23	0.845	292.34	92.16	60.36	0.808	0.887	7^a^
Equation 24	0.821	300.42	87.25	62.14	0.805	0.884	28
Equation 25	0.835	274.00	88.24	63.57	0.778	0.865	11
Equation 25a	0.835	269.89	88.24	63.57	0.793	0.876	12
Equation 26	0.822	296.12	89.22	59.29	0.793	0.876	26
Equation 27	0.845	330.75	91.18	62.14	0.780	0.865	5^a^
Equation 27a	0.845	325.79	91.18	62.14	0.805	0.885	6^a^
Equation 28	0.827	291.93	88.24	63.21	0.805	0.885	19
Equation 29	0.825	290.35	89.22	59.64	0.785	0.869	22
Equation 30	0.845	282.34	92.16	60.36	0.782	0.868	8^a^
Equation 31	0.847	296.16	93.14	60.00	0.805	0.884	3^a^
Equation 32	0.846	297.08	93.14	60.00	0.808	0.886	4^a^
Equation 33	0.844	299.50	91.18	62.50	0.805	0.884	9^a^
Equation 34	0.841	303.35	87.25	64.29	0.801	0.882	10^a^
Equation 35	0.786	297.85	81.37	62.50	0.738	0.834	32

*Note*

a Ten equations with very tightly similar AUCs: ranging from AUC 0.849 (Equation 20) to AUC 0.841 (Equation 34).

### 3.4 Feature selection

**Figure 1:**
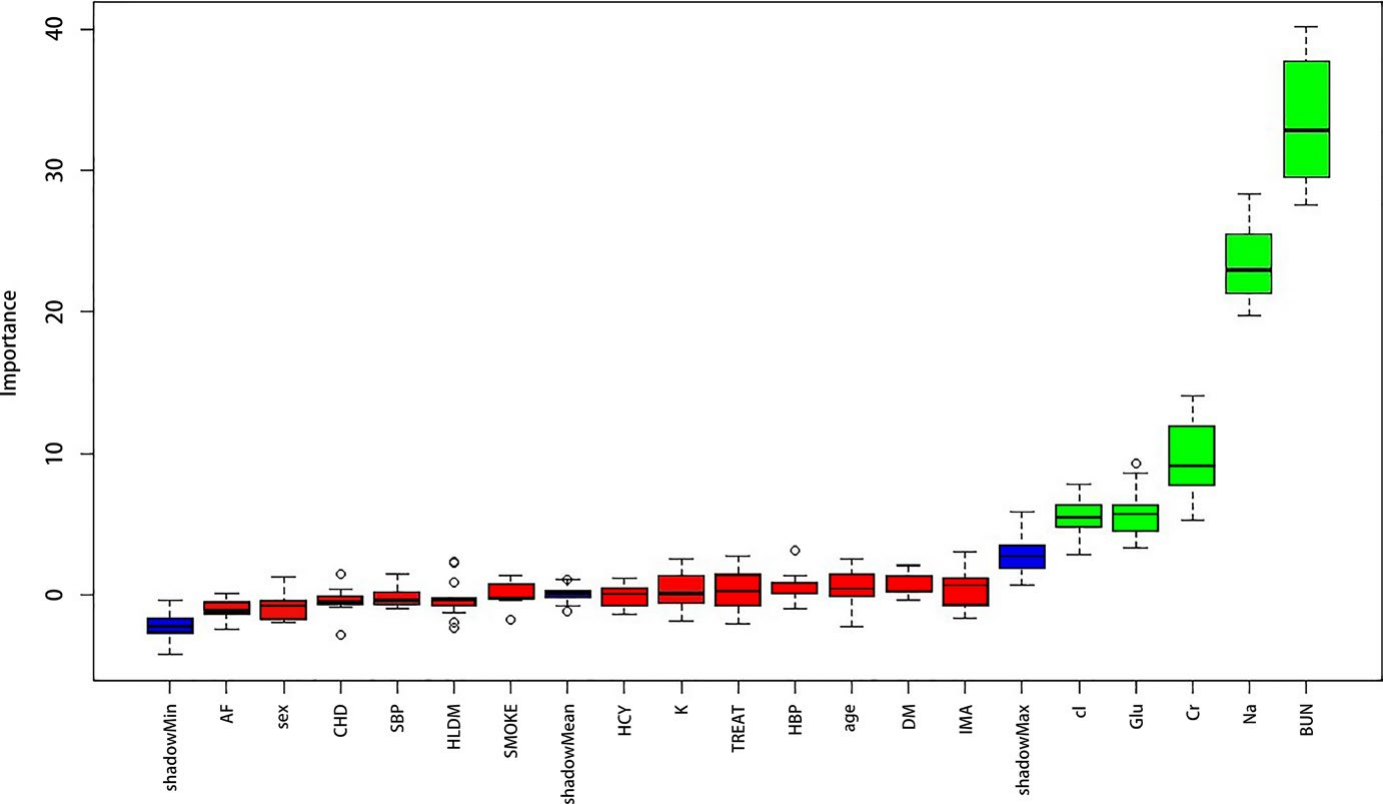
Five attributes confirmed important: Cr(creatinine) + BUN(serum urea) + Cl(chlorinum) + Glu(glucose) + Na(sodium)

There were 18 input variables, which included personal information (gender, age, and vascular risk factors), laboratory studies (serum sodium, potassium, chlorinum, glucose, and urea). The Boruta algorithm was applied to select the most characteristic structural patterns. Five variables were confirmed to be important: creatinine (Cr)  + serum urea (BUN)  + chlorinum (Cl)  + glucose (Glu)  + sodium (Na). Superfluous parameters were removed; these 13 variables that were confirmed to be unimportant were atrial fibrillation (Af), age, coronary heart disease (CHD), diabetes (DM), hypertension (HPT), and 8 others. These results are shown in Figure 1.

### 3.5 Optimized SVM parameters

Before processing the SVM model, the penalty parameter “cost” and kernel function parameter “gamma” were optimized using the tune () function. The results listed the sampling method (10‐fold cross‐validation), the best parameters (gamma = 0.001, cost = 64), the best performance (0.166), and the details of the tested parameter values.

### 3.6 ROC curve for ND prediction with an SVM model

**Figure 2:**
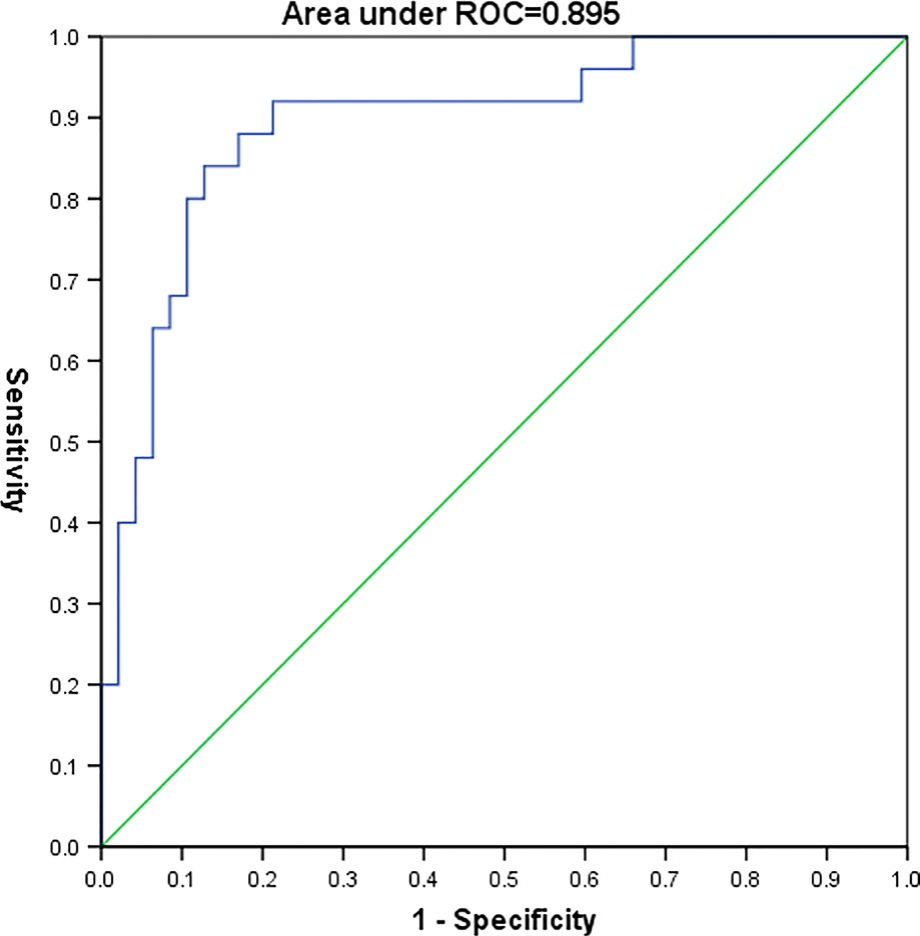
The ROC curve (raw data)

**Figure 3:**
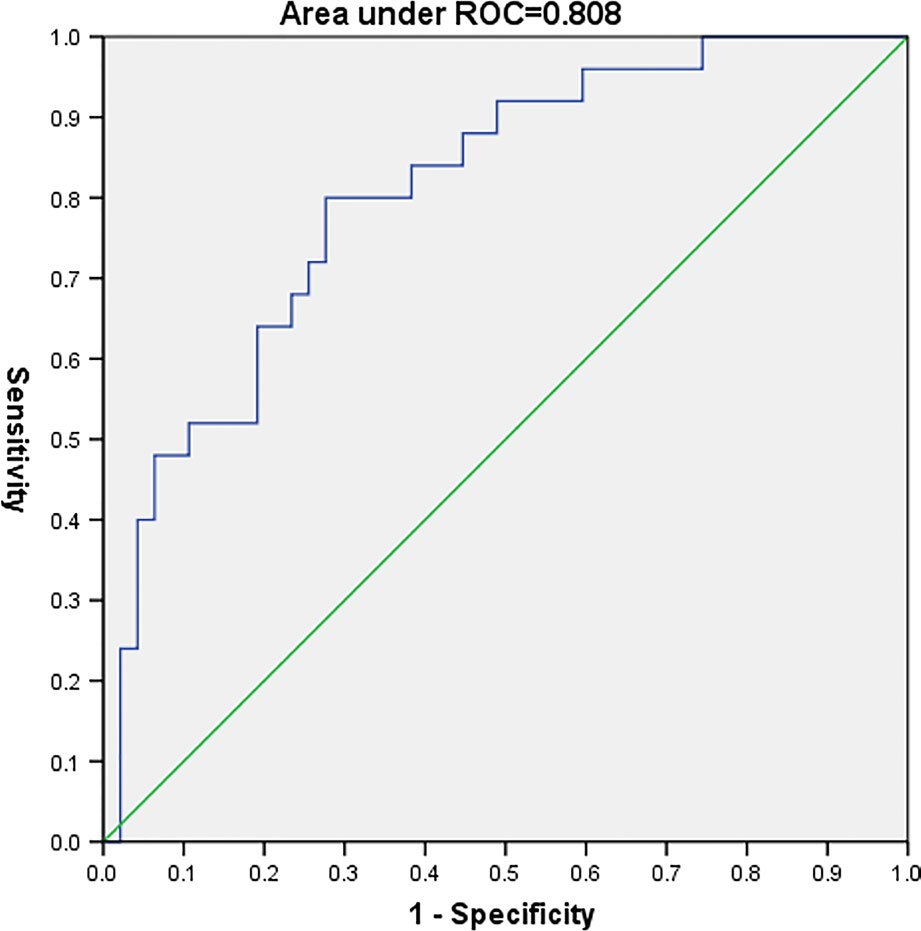
The ROC curve (selected data)

We used two SVM classifiers, using the raw data and feature‐selected data after applying the Boruta algorithm, respectively. The results are shown in Figure 1. Sensitivity, specificity, and AUC based on ROC analysis were used to assess the performance of classifiers. Figures 2 and 3 show the ROC curves for the classifiers applied to the different datasets. The AUC values for the different classifiers were 0.700, and 0.927 for the raw data and feature‐selected data, respectively. The classifier applied to feature‐selected data was effective in distinguishing the two categories of patients. The performance of the classifier on feature‐selected data after applying the Boruta algorithm had higher sensitivity and specificity than that applied to the raw data. The cutoff values with the highest sensitivity and specificity were 0.272 for feature‐selected data (sensitivity = 92.0%, specificity = 89.4%) and 0.155 for raw data (sensitivity = 93.8%, specificity = 44.1%).

## 4 DISCUSSION

In this study, we ranked 38 equations with indicators of dehydration according to the AUC values for predicting ND in poststroke patients; Equation 20 (1.86 ×  Na+ + glucose + urea + 9, with all components measured in mmol/L) had the highest performance for prognosis of ND among all indicators of dehydration. There are similarities across these three studies (Heavens et al., 2014; Hooper et al., 2015; Siervo et al., 2014), with some equations appearing in more than one study. Equation 32 appeared to work well for predicting serum osmolarity in older as well as younger adults and for predicting poststroke ND. Given that this single equation works well across all situations and population groups, this may be the appropriate equation to use for serum osmolarity. However, the SVM model based on feature‐selected data yielded even better predictive accuracy than Equations 20 and 32.

Determining the pathophysiological mechanism of ND occurrence was beyond the scope of this study. However, there are some possible explanations. Increased serum osmolarity can be attributed to increased serum glucose levels (hyperglycemia) in patients with acute ischemic stroke (Bhalla et al., 2000). The evolution of hyperosmolality and hypernatremia has been observed in ischemic stroke patients, but the level of potassium appears to remain stable (Natochin Iu et al., 1996). Those findings were in keeping with our results.

A dehydrated status might worsen intravascular volume depletion (Lang, 1987), affecting oxygen delivery and proper organ function, which may contribute to the development of ND (Weinberg et al., 1994). Hyperosmolality could also trigger DNA damage, cell cycle arrest, mitochondrial depolarization, protein carbonylation, mitochondrial depolarization, cell shrinkage (Brocker, Thompson, & Vasiliou, 2012), and oxidative stress (Faraco et al., 2014).

In our study, hypertension, diabetes, hypercholesterolemia, ischemic heart disease, and smoking were considered as vascular risk factors in the analysis. The baseline characteristics between the patients with ND and without ND were not significantly different.

In laboratory tests, participants with ND had higher BUN, creatinine, and fasting plasma glucose concentrations. However, these indicators were not abnormal. This means that these indicators could potentially play a role in dehydration. These findings were similar to those of previously published studies (Bhatia, Mohanty, Tripathi, Gupta, & Mittal, 2015).

This study compared the BUN/Cr ratio and 38 equations incorporating dehydration parameters for predicting ND by means of an ROC curve. Equation 20 most accurately predicted ND in patients with acute ischemic stroke, but was not yet perfectly accurate, with a sensitivity of 94.12% and a specificity of 61.43%; low specificity affects predictive accuracy. Equation 32 had a sensitivity of 93.14% and specificity of 60.00%, while the BUN/Cr ratio had a sensitivity of 81.37% and a specificity of 67.5%.

The cutoff value for the BUN/Cr ratio that could maximize the sum of sensitivity and specificity was 20.66, which higher than the value of 15 used in previously published studies (C. J. Lin et al., 2016; L. C. Lin et al., 2014; Rowat et al., 2012; Schrock et al., 2012). The mean BUN/Cr ratios in patients with ND and without ND in our study were both higher than 15. These differences in cutoff values between our study and previous studies may be due to differences in reagent and measurement methods. Nevertheless, it is clear that an increase in the BUN/Cr ratio is associated with ND.

In addition, before training the SVM model, we used the tune () function to select appropriate penalty parameter (cost) and kernel function parameter (gamma) values. If these values were inappropriate, overfitting or underfitting may have occurred (Reid et al., 2010). Parameter optimization is important to improve the precision of classification. Because the RBF has been proven to make the kernel function more compatible, we used RBF as a kernel function for training (Cawley & Talbot, 2004). RBF also reduced the computational complexity and improved the generalization performance. The Boruta algorithm could capture all features that were either strongly or weakly relevant to the outcome variable. As compared to the traditional feature selection algorithm, the Boruta algorithm more appropriately identifies variables of importance (Blog, 2016). Consequently, it is well suited to biomedical applications. We adopted the Boruta algorithm to reduce the number of variables from 18 to 5. The specificity of allocating test samples after this process was increased from 44.1% to 89.4%, and the AUC was increased from 0.700 to 0.927. Thus, the accuracy of classification was increased by implementing the Boruta algorithm.

Although the sensitivity of Equation 20 was higher than that of the SVM model, it still had the limitation of a low specificity. Low specificity could lead to many false‐positive and false‐negative results and make clinical prediction uncertain. Nevertheless, the AUC of the SVM model was significantly higher than that of Equation 20. As the AUC should be maximized to obtain the best possible prediction method, the SVM model represented the best predictive accuracy.

Several limitations of this study need to be considered. First, the generalizability of the results may be limited, as it was a single‐center study. Second, this study lacked a serum osmolarity measure of dehydration to serve as a gold standard for comparison. Third, our data suggested that an SVM model incorporating dehydration measures would be able to predict ND, but it is necessary to assess its effect on different stroke subtypes. Fourth, patients with reperfusion therapy were not included in this study. We also did not assess outcomes beyond 3 months and did not consider mortality. A follow‐up of longer than 3 months would allow evaluation of the long‐term predictive accuracy of this approach.

In conclusion, dehydration, which is common in hospitalized stroke patients, was associated with ND at the hospital. High‐level machine learning techniques (SVM) were used to establish a prediction model for ND associated with dehydration, and achieved good classification results. Feature selection by the Boruta algorithm can eliminate redundant features, biases, and unwanted noise and can increase prognostic accuracy. The tune () function can be used for SVM model parameter optimization to improve the results. It could be helpful for detecting patients with dehydration in order to predict and prevent ND. The application of SVM in this study provides a basis for designing and more efficient data analysis. In future studies, other dehydration information can be integrated into this process to improve the precision of classification.

## CONFLICT OF INTEREST

None declared.
